# Offense-Related Issues in Forensic Psychiatric Treatment: A Thematic Analysis

**DOI:** 10.3389/fpsyt.2019.00925

**Published:** 2020-01-08

**Authors:** Riitta Askola, Päivi Soininen, Allan Seppänen

**Affiliations:** ^1^Division of Mood Disorders, Helsinki University Hospital, Helsinki, Finland; ^2^Department of Nursing Science, Faculty of Health Sciences, University of Eastern Finland, Kuopio, Finland; ^3^Psychoses and Forensic Psychiatry, Helsinki University Hospital, Helsinki, Finland; ^4^Vanha Vaasa Hospital, Vaasa, Finland

**Keywords:** offense, forensic psychiatry, rehabilitation, risk, good lives model

## Abstract

Characteristics unique to forensic psychiatric treatment include coming to terms with the offenses committed, the long duration of treatment and the assessment of the risk of repeat offending. This study describes the views of both patients and staff on the significance of the patient’s offense as a part of forensic psychiatric rehabilitation. Eight forensic psychiatric patients and eight forensic psychiatric nurses from two forensic psychiatric hospitals in Finland participated in this study. Data were gathered by means of thematic interview and analyzed by means of thematic analysis. The findings suggest that patients and professionals alike concur that ascertaining the factors with a bearing on the offense, and working through the offense and the factors leading up to it, constitute an essential aspect of forensic treatment. This, in turn, has a bearing on the planning and administration of a treatment plan consisting of both medical and psychosocial support and interventions intended to enable patients to live independent, fulfilling lives, thus reducing the likelihood of reoffending. The findings of this study can be used as part of the development of international, standardized treatment models for clinical forensic psychiatric practices.

## Introduction

Practicing forensic psychiatry requires special legal and criminological knowledge, clinical skills and experience of treatment of often complex and coexistent mental disorders (1). The Joint Commissioning Panel for Mental Health (2) defines the remit of forensic psychiatric services as follows: forensic mental health services for individuals a) with a mental disorder (including neurodevelopmental disorders) b) who pose, or have posed, risks to others, and c) in the case of which that risk is usually related to their mental disorder.

Despite these common denominators, forensic patients are a heterogeneous group in terms of the details of their offense history, psychopathology and risk factors (3). That notwithstanding, in most jurisdictions, forensic psychiatric patients suffer primarily from disorders with psychotic symptomatology, but comorbidities are very common, especially personality disorders, neurodevelopmental disorders and substance-related disorders (4–6).

### Index Offense

Patients who end up developing an offending history have typically experienced 7–8 inpatient episodes in general psychiatric care before committing their index offense and receiving subsequent treatment as forensic patients (7). More than three-quarters of forensic patients have previously been admitted to general psychiatric care and nearly 40% have committed offenses before their first admission to general psychiatric treatment (8). In 2019, the most common offenses for which people were examined in a court-ordered forensic assessment in Finland were homicides (30%) and other violent acts (40%), while others were mainly arson, sexual offenses, and crimes against property (9).

Working through the offense in a supportive and therapeutic relationship is an important part of reaching the goals of forensic care; it entails on the one hand a causal exploration of how prior events, situational factors, and choices contributed to a particular offense. On the other hand, it is also a dynamic process by which the offense is integrated into the patient’s life narrative in an attempt to move beyond it. If the offense is not worked through, the risk of reoffending will remain (10–16) and the patient offender may see his offense as an absolute, identity-defining act, from which there is no conceptual or moral escape. Therefore, in order to develop an understanding of the dynamics of offending, the events, circumstances, and behaviors that occurred before, during and after the offense should be analyzed (14). The process of working through the offense can be approached in various ways, depending on the patient’s distinctive responsivity, behaviour, and style of interaction (17). The approaches are as follows: 1) increasing the psychotic patient’s sense of security, 2) building trust with a suspicious patient, 3) understanding a defensive patient’s behavior, 4) discussions of thoughts, impressions, and emotions with a patient facing reality, 5) increasing support and caring for the depression of the patient working intensively. When successful, this process of mutually responsive interaction will eventually lead to the integration of the offense into the patient’s life experience. Naturally, these approaches are not mutually exclusive: the patient will typically respond differently according to the therapeutic stage he has reached and may even require several approaches simultaneously while moving forwards—and sometimes backwards—in the clinical process (17).

### Forensic Psychiatric Assessment and Treatment

Forensic treatment must be able to mitigate the risk of reoffending by affecting and intervening in, among other things, psychotic symptoms and impulsivity (18). Accordingly, one of the key issues that distinguish forensic services from general psychiatric services is the central role of the legal framework in which assessments, clinical processes, and decisions take place (19–21); thus forensic services have a pronounced dual commitment, bound on the one hand by patient-centred medical ethics, and on the other by legal stipulations (1). As an example, risk assessments are required both in the context of providing expert evidence to the courts and in the planning of treatment interventions. This, of course, necessitates reliable and valid risk assessments to assign individuals to treatment programs based on the risk they pose (1, 22)

To this end, current forensic practice uses structured professional judgement (SPJ) assessment tools that consider static risk variables and dynamic, modifiable variables, thus presenting forensic professionals with potential utility in treatment planning and implementing specific treatment programs (1). Among the best known treatment programs are the Risk-Need-Responsivity model (RNR model) and the Good Lives Model (GLM). The RNR model is based on the three primary principles of risk, need, and responsivity and their associated assumptions (1, 3, 23). The first two principles (risk and need) are used to determine treatment intensity and targets and the whole set of principles is employed to guide the actual implementation of treatment (24). The Good Lives Model, on the other hand, is a strength-based approach to offender rehabilitation (25) in which risk factors are viewed as obstacles that erode individuals’ capacities to live more fulfilling lives (26, 27). It emphasizes offenders’ personal preferences, values, and goals, drawing upon this understanding to motivate them to live better lives. It also equips offenders with the capabilities and resources to obtain so-called primary goods in socially acceptable ways (28–31). According to Rawls (28) the primary social goods are rights, liberties, and opportunities, and income and wealth and a sense of one’s own worth. In a clinical context, these primary goods were further explored and sub-defined by Purvis et al. (29) as: “1) life (including healthy living and functioning), 2) knowledge (how well-informed people feel about things that are important to them), 3) excellence in play (hobbies and recreational pursuits), 4) excellence in work (including mastery experiences), 5) excellence in agency (autonomy and self-directedness), 6) inner peace (freedom from emotional turmoil and stress), 7) relatedness (including intimate, romantic and familial relationships), 8) community (connection to wider social groups), 9) spirituality (in the broad sense of finding meaning and purpose in life), 10) pleasure (the state of happiness or feeling good in the here and now) and 11) creativity (expressing oneself through alternative forms)” (29). In this theoretical framework, criminal behavior occurs when individuals lack the internal and external resources necessary to satisfy their values using pro-social means (29). According to Lord (32), the GLM appears to provide a better fit for the recovery needs of forensic patients than the Risk–Need–Responsivity model because it emphasizes approach goals, enhanced responsivity, and skills acquisition.

The possibility of constructing and translating conceptions of good lives into actions and concrete ways of living depends crucially on the possession of internal (skills and capabilities) and external (opportunities and supports) conditions (26, 27). In institutional conditions, the latter is largely affected by the quality of the therapeutic relationships between the patient and professional staff. Once a well-functioning therapeutic alliance has been established, attention can be paid, for example, to what prompts the substance abuse behind the offense (*e.g.* loneliness, lack of meaningful activity, antisocial peer group) and an attempt can be made to influence emotional regulation and behavior, and to try to get the patient to focus on more constructive behavioral models. This work requires significant therapeutic support, and the named nurse has a particularly crucial role throughout the process. By displaying high levels of empathy and understanding, the named nurse must lay the groundwork for the therapeutic alliance and counteract the feelings of defeat and entrapment so often experienced by patients under compulsory psychiatric treatment (33), particularly after having committed a self-traumatizing offense.

This study describes the views of both patients and staff regarding the significance of the patient’s offense as a part of forensic psychiatric rehabilitation. We highlight this particular aspect of forensic rehabilitation, within the conceptual framework of GLM, as an essential element of the care and risk-management of forensic psychiatric patients.

## Methods

### Participants

Eight forensic psychiatric patients (seven men and one woman) and eight forensic psychiatric nurses from two forensic psychiatric hospitals in Finland were interviewed. The inclusion criteria for the patients were: 1) age over 18 years, 2) has committed an offense, 3) mentally stable enough to participate (*i.e.* no excessive anxiety anticipated due to participating), and 4) sufficient proficiency in Finnish. The exclusion criteria were mental instability (acutely psychotic, suffering from anxiety, likely to self-harm, or in the personnel’s estimation likely to be adversely affected by participating in the proposed study). The patients, aged 30–50, were inpatients (n = 6), and outpatients (n = 2) discharged by the National Institute for Health and Welfare (THL) under supervision and living in psychiatric rehabilitation units. The offenses included homicides (four patients), crimes against property (one patient), assaults (two patients) and arson (one patient) (**Table 1**). The inclusion criterion for forensic psychiatric staff participant selection was being a registered nurse (RN) or mental health nurse with experience in therapeutic approaches to forensic patients’ criminal offenses in a nurse–patient relationship, including acting as a named nurse. The sample selection was based on the relevance of the nurses’ experience, with all nurses selected having at least 10 years’ psychiatric nursing and 5 years’ forensic psychiatric nursing experience (**Table 2**).

**Table 1:** Demographics of the patients.

Patients	Gender	Age	Status	Index offense	Interviews
P1	Male	30	Inpatient	Assault	1
P2	Male	35	Inpatient	Homicide	1
P3	Female	46	Outpatient	Homicide	1
P4	Male	44	Inpatient	Assault	1
P5	Male	38	Inpatient	Arson	1
P6	Male	38	Inpatient	Homicide	3
P7	Male	36	Inpatient	Crimes against property	3
P8	Male	50	Outpatient	Homicide	3

**Table 2:** Demographics of the nurses.

Nurses	Gender	Age	Education
N1	Female	59	Mental health nurse
N2	Female	45	Mental health nurse
N3	Male	37	Mental health nurse
N4	Male	43	Mental health nurse
N5	Male	40	Registered nurse
N6	Male	38	Registered nurse
N7	Male	48	Mental health nurse
N8	Female	36	Registered nurse

### Procedure

Ethical approval for the study was obtained from the Ethics Committee of the Hospital District of Helsinki and Uusimaa. Formal approval and permission for data collection in Finnish psychiatric hospitals with forensic psychiatric patients were granted. Having obtained permission for data collection the researcher informed nurse managers and the staff on the wards and out-patient-clinics about the study. The staff suggested suitable patients whom they thought would not be distressed by the study and who were in a stable enough condition to participate in relatively lengthy interviews. Participants were given written and verbal information regarding the study and formal informed consent was obtained from all participants.

The researcher (RA) conducted all the interviews herself as thematic interviews, in which the participants were asked to describe the offense and issues related to it. Thematic interview was chosen as a method, as it allows acquiring qualitative information about a topic or about a field which is relatively less known or rarely studied. It focuses on subjective experiences as defined and narrated by the interviewees, and accepts this as valid material for scientific scrutiny (34, 35). The researcher was not previously known to the nurses or the patients interviewed.

All interviews with patients were individual interviews lasting from half an hour to 2 h (mean 90 min) and progressed by unstructured discussion of their offense, and how they had dealt with the possible feelings it raised. Only the participant and the researcher were present during the interviews. Three patients were met three times at their request, with each interview session lasting 2 h, due to their wish to go over their experiences in detail, others were met once. All interviews were recorded except one, as the patient objected. The researcher took notes instead.

Six individual interviews with nurses and one interview with two nurses were conducted. The unstructured interviews lasted 1 to 2 h (mean 90 min) and progressed informally by discussing the topic and different patient cases. All interviews with nurses were recorded. All interviews were transcribed verbatim. A total of 162 pages of material (1.5 spacing) resulted. Of these, 98 pages concerned the interviews with patients and 64 pages the interviews with nurses.

### Analysis

The data were analyzed by using inductive thematic analysis. Thematic analysis is a method for identifying, analyzing, and reporting patterns (themes) in data (36) and a process of interpretation of qualitative data to identify patterns of meaning across the data (37). Thematic analysis is particularly suitable for analyzing subjective experiences, perceptions, and understandings (38, 39) and a rigorous thematic analysis can provide trustworthy and insightful findings (36, 40).

The patients’ and the nurses’ data were analyzed together, as according to Braun and Clarke (36) thematic analysis is a useful method for examining the perspectives of different research participants, highlighting similarities and differences, and generating unanticipated insights.

Inductive analysis is a process of coding the data without trying to fit it into a pre-existing coding frame or the researcher’s analytic preconceptions (36). In this sense, this form of thematic analysis is datadriven (36). Coding and theme development as steps in thematic analysis are driven by the goal of retaining considerable detail in the data items (39).

The data were analyzed in six phases (36). First, the researcher made herself familiar with the data by listening to the audiotapes and reading the transcripts several times to develop a thorough understanding of them. Second, the researcher generated initial codes from the data which identified a feature of the data that appeared noteworthy to the analyst. The entire data set was organized into meaningful groups according to the codes. Codes are labels applied to segments of data which are likely to be relevant in the context of the research questions (39). Coding was done manually and no qualitative data analysis software was used in analyzing the data. The researcher used line-by-line coding to code every line to open up the data. After that, the codes were sorted into potential themes, with consideration of how codes may combine to form an overarching theme. In the fourth phase, the researcher checked that the themes worked in relation to the coded extracts and to the data as a whole. A candidate thematic map of the analysis was generated. After that, the themes were defined and named and sub-themes identified. The researcher again checked the coherence of the themes and each theme in relation to the others. In the sixth phase the report was produced after selection of vivid, compelling extract examples. The researcher ensured that these related to the research question and the literature.

## Results

**Figure 1:**
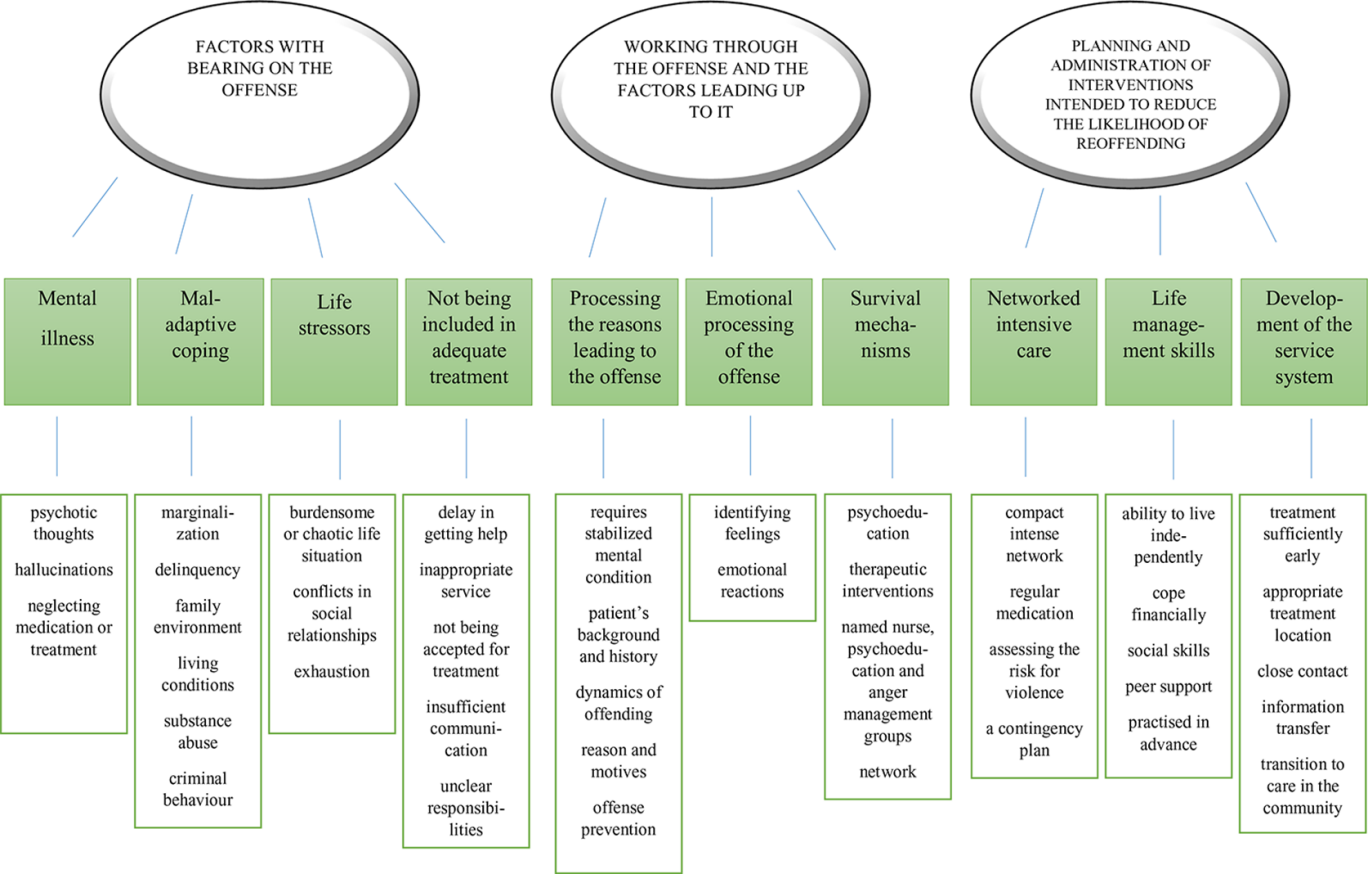
Map of thematic analysis.

Three main themes, each with sub-themes, emerged: 1) the factors with a bearing on the offense, 2) working through the offense and the factors leading up to it, and 3) the planning and administration of interventions intended to reduce the likelihood of reoffending (**Figure 1**).

### Factors With Bearing on the Offense

This main theme contained the sub-themes mental illness, maladaptive coping, life stressors, and not being included in adequate treatment.

#### Mental Illness

Both patients (P) and nurses (N) described the major significance of mental illness as a reason leading up to the offense. Psychotic thoughts or hallucinations may have had violent, threatening or frightening content and possibly exhortations to act violently. At the time of the offense, patients did not for the most part understand that they were mentally ill, nor did they know how to cope with their symptoms or where to seek help. One patient described how she had led a kind of double-life, whereby she maintained a facade of normality. Her psychiatric illness, and the resultant homicide, came as a complete surprise to her family.

I pretended that everything was fine. They didn’t know about this. When I was arrested my mother knew nothing about anything. I never went home to my parents when I was messed-up, I never took my mates there. This came as such a surprise to both of them. I had another life in that crew. It was a real shock to them. I was in prison, and my dad was completely … my mum did visit me. P3

First I got these auditory hallucinations and then visual hallucinations, dead people telling me all kinds of things. P2

My thoughts are so very unrealistic, so distressing, violent, brutal, sick in many ways. P3

The patient may have neglected medication or treatment, which exacerbated their psychotic state and led to the offenses being committed. Active substance abuse may also have been a cause for neglecting the treatment of mental illness.

I didn’t treat my illness in any way at all. Just the opposite. I just got worse and worse by doing drugs. P3

#### Maladaptive Coping

Several patients had a history of social or socioeconomic marginalization or delinquency. According to both patients and nurses, the quality of the family environment and living conditions may also have been insufficient, dismal, even violent, and the adaptation and coping strategies weak. Nurses described how adverse childhood experiences caused challenges in evoking trust in the treatment relationship.

It’s great if you even get to the point where you can discuss the offense. It really is quite difficult to achieve. It’s often the case, that there has never been any trust. No-one to trust since childhood, and no trust in those circles where they’ve hanged out, and having experienced prison and what not. Then to start building trust towards a nurse you’ve never met … N3

Often the foundations of life have been so bad—poverty, dreadful family background—that it feels normal to be sometimes knocked about and so on. N3

The patients’ substance abuse or criminal behavior may have begun very early. Patients described the difficulty of stopping substance abuse (drugs, alcohol, steroids) and a criminal lifestyle. Some of the patients had been in prison several times.

I started doing stupid things when I was about seven and so it gradually started, setting fire to rubbish, just senseless things. Then year by year they got worse and worse, the things I did, and then it was prison. A few times in prison. P6

Three men, drinking, and one gets killed. You only need to walk into somebody and that’s it. P8

#### Life Stressors

Some of the participants described that a burdensome or chaotic life situation contributed more to the offense than mental illness. Life stressors affecting mental coping that were mentioned included conflicts in social relationships and exhaustion.

With this one patient it was down to jealousy and he’d killed his girlfriend. N2

It was exhausting, being alone with a child. N1

#### Not Being Included in Adequate Treatment

The patients had plenty of experience of not getting adequate treatment and being excluded, which contributed to the commission of the offense. Examples included delay in getting help, being directed to an inappropriate service or not being accepted for treatment. Communication between authorities (police, healthcare professionals and social welfare authorities) was insufficient and areas of responsibility were unclear. One patient described how psychiatric services redirected him to substance misuse services, which, in turn, neglected to treat his delusional psychosis. A week later, he set fire to a residential building.

I’d got out of hospital like a week earlier. They didn’t give me any pills, nothing. It’s like malpractice on their part. P5

### Working Through the Offense and the Factors Leading Up to It

This main theme contained the following sub-themes: processing the reasons leading to the offense, emotional processing of the offense, and strengthening the patient’s survival mechanisms.

#### Processing the Reasons Leading to the Offense

Both the patients’ and the nurses’ view was that analyzing the determinants of the offense is not possible until a patient’s mental condition has been stabilized and it is clear that the patient’s mental state can withstand the processing. The cyclical process comprises numerous steps and phases while observing the patient’s mental condition. The nurses said they took a lot of time to examine the patient’s background and history. In doing so, they tried to understand the dynamics of offending and to plan relevant intervention strategies.

It took something like two to three years there in the hospital before it started getting easier and I was better able to process the issue and go through it. P2

The named nurse is very sensitive in that respect, in taking things forward in very small steps, listening for our feelings about where we are, so the patient is under the control of the whole group after these discussions have been held. We know that there’s a risk of something happening. N8

The nurses said that they tried to identify the patient’s thoughts, impressions, emotions, and psychotic delusions or hallucinations to understand the reason and motives for the offense.

At the beginning, I wouldn’t really express my own impressions, but, rather, we’d go through his points of view. Then, by small steps, could this or that be the reason why you are here now, because his own interpretation might not be totally realistic. N8

How had the patient’s thoughts gone in that direction and been unrealistic, how had the patient misinterpreted them, and what would he think now? Because the patient had received signs which had led to the interpretation that his spouse was cheating. Seek an explanation and if there is none, then … N2

The participants analyzed how the offense committed could have been avoided. Seeking help earlier and discussing their situation with a professional were mentioned, but patients who had served prison sentences, in particular, said that attitude and lifestyle are crucial.

I should finally have gone to the doctor or to the health centre or done something, told some professional about those feelings and thoughts and delusions. P2

When you’re younger there’s this principle that you get through it by doing time, such attitudes and thoughts have changed along the way, and every person and animal should be allowed to live life, nobody should take that away from anyone else. P8

#### Emotional Processing of the Offense

Feelings, such as anxiety, guilt, shame and suicidal thoughts, arose in the patients when working through their offense. Both patients and nurses considered identifying these feelings important. According to both patients and nurses, working through the offense may be emotionally frightening and distressing for the patient.

This processing of feelings and owning them, I find it has been difficult for our patients in almost all treatment relationships. N8

It was also possible that the desired emotional reaction was not evoked. If the patient is suspicious and hostile it is important to identify the patient’s defense mechanism and not to pressurize him. The patient may blame other people, even the victim, or even refuse to work through his history and the offense. One patient described working through the offense as so emotionally distressing, and the offense so shameful, that his condition was much better when the offense was not talked about.

(Patients) deny this entire institution and in a way accept that their acts are justified, for example killing your wife—some even think that’s justified. They don’t accept treatment at all as a form of care. It’s taken us six years with this, nothing … it’s this constant denial, making no concessions however hard you try. And the risk of repeating is still so high. N5

He stole my smokes, he did wrong by me, and I punished him for it. P1

The nurses described attempting to evoke normal emotional reactions, like guilt and empathy, but it was seen as important not to do this by means of emotional pressure through castigation or moralization. Rather, working through the offense required a neutral tone from professionals to fully support the emerging emotions in patients.

In the treatment relationship we have considered whether the patient thought about what family members would think and feel about this. Whether the patient is able to put himself in the other person’s shoes and think what the other person might be feeling. N8

#### Strengthening Survival Mechanisms

By using psychoeducation and therapeutic interventions, the patient’s coping may be strengthened, and self-awareness of the mind and its workings increased. Also, both patients and nurses agreed that peer support was invaluable in presenting positive examples of treatment progress.

We just finished the psychoeducation group yesterday; it left me feeling really good. I got a lot of info and people talked about their experiences and thoughts, what has happened, and there was peer support too. The things people have gone through before arriving at this point. P2

In psychoeducation we have made use of peer support. We talk about schizophrenia, what it is and what it involves. N5

The patients felt they got help from their named nurse, psychoeducation and anger management groups, which gave them tools and methods to control their aggression, anger and temper. It is very important to identify and control the emotional triggers which led to the offense so that one can intervene in time and so that in the future the situation does not reach the same point at which the offense occurred.

I’ve learned to know my own head. Last spring, there was this thing about controlling violence, I think it lasted about half a year, I attended that. P8

He still has violent escape thoughts and in these things those offenses under prosecution come up. Then you think that he has committed serious acts and still has such visions and thoughts of absconding, then he could do the same thing again. N7

Close contact between the patient and his or her network is significant. The situation was more sensitive when the offense had been committed directly against a family member or the members of the family did not want to meet the patient. The nurses said they always observed the potential risk the patient might pose to his family or network.

There’s a religious element involved in this: to make amends with God he had sacrificed his family and still from time to time he toys with such thoughts. N4

### Planning and Administration of Interventions Intended to Reduce the Likelihood of Reoffending

This main theme contained the sub-themes of networked intensive care, life management skills and further development of the service system.

#### Networked Intensive Care

All the participants emphasized the significance of a compact and intense network for attempting to reduce recidivism. Contacts between the multiprofessional team and the patient’s relatives and close friends are important, as are those between services and authorities which support the patient after discharge. The network was felt to be important both in terms of supporting self-esteem and providing everyday encouragement, and in providing help when encountering concrete difficulties.

It’s a good thing that there’s a safety network, it’s important, having such people around you. That there’s AA, family, relations, friends, all up and running. I have to say that this is something of a survival story. P3

When I’m sober I have no problems with violence control, but if I take a drink, out come the pent-up issues of twenty years. I go to the AA club once a week. P8

Both patients and nurses considered regular medication crucial in minimizing recidivism: the patient’s motivation for regular medication as prescribed, and the regular evaluation thereof, were viewed as essential.

I consider medications really important; I am sure to be taking those medications for the rest of my life so nothing like this will happen in the future and so I can live what you would call a normal life and cope with this illness in the future, too. P2

According to the nurses, repeatedly assessing the risk for violence is also important in follow-up care after treatment in hospital. Identifying burdensome and triggering factors, on the one hand, and strengthening, protective, life-management reinforcing factors, on the other, was seen as key in stabilizing both pre- and post-discharge treatment.

I certainly keep an eye out for signs that a patient may be losing control, and I need to keep that in mind and evaluate, and while he may have done nothing I have to be aware that he could do it for the first time now. N7

According to both patients and nurses it was important to draw up a contingency plan for the event of a destabilizing situation to be able to decrease the likelihood of reoffending. Although individualized, plans typically included avoiding contact with substance abusers, having hobbies and avoiding too much time alone. The patients interviewed had also thought about what they might be able to tell other people about themselves and their illness. The role of a forensic psychiatric patient undergoing rehabilitation might be found to be stigmatizing and this theme was processed in advance during hospital treatment, before going back into the community.

I no longer hang out in those circles where they do drugs. I stay away from those so I don’t have to talk with them. You know what that would lead to. P7

#### Life Management Skills

According to both patients and nurses, it was important to promote skills in daily living and rehabilitation and to support patients’ independence and life management, which in turn would enhance their self-esteem. Over a lengthy hospital stay, many skills for daily living had deteriorated, and it was important to revive these so that post-discharge daily life would be smooth and not provoke helplessness and frustration. According to the interviewees, supporting patients’ ability to live independently and cope financially, and their general social skills, was important. They also emphasized the importance of peer support.

Maintaining activity, social skills. It’s important and something we often forget when patients go on somewhere out of the hospital. Patients say that two years in hospital makes a person pretty incapable of normal functioning. N6

My biggest plan is a place of my own to live. Then I would like some sort of … I have thought of some sheltered type of work or part-time work, then you’d have a rhythm in your life so that you could break your day into parts and then get some human contact. Then hobbies, exercise, going for walks. P2

Skills for everyday life were practiced in advance in the safe-environment of the hospital or on making the transition to post-discharge life.

One has to learn everything all over again, and then you realize that I can do this, I can see myself return to society, I have money in my hand, I can pay, I can get back change. N5

I’ve been practising in an outpatient clinic for some time now, I come here for blood tests and to talk to the doctor and to get my pill dispensers. P5

#### Further Development of the Service System

The patients and nurses interviewed voiced their views on the further development of the psychiatric service system. They saw initiating treatment sufficiently early, referral to an appropriate treatment location and close contact after hospital treatment as important issues. Ensuring information transfer between services and authorities and a clear definition of the duty of care are salient for treatment success.

I tried to get into hospital but they wouldn’t admit me because my alcohol intoxication level was 1‰. P4

It emerged in the interviews that the transition to care in the community must take place sufficiently slowly, in a controlled and gradual manner.

Then the treatment progresses, and the circles extend a bit outside the ward, various problems arise, or challenges which need to be processed. N8

## Discussion

**Figure 2:**
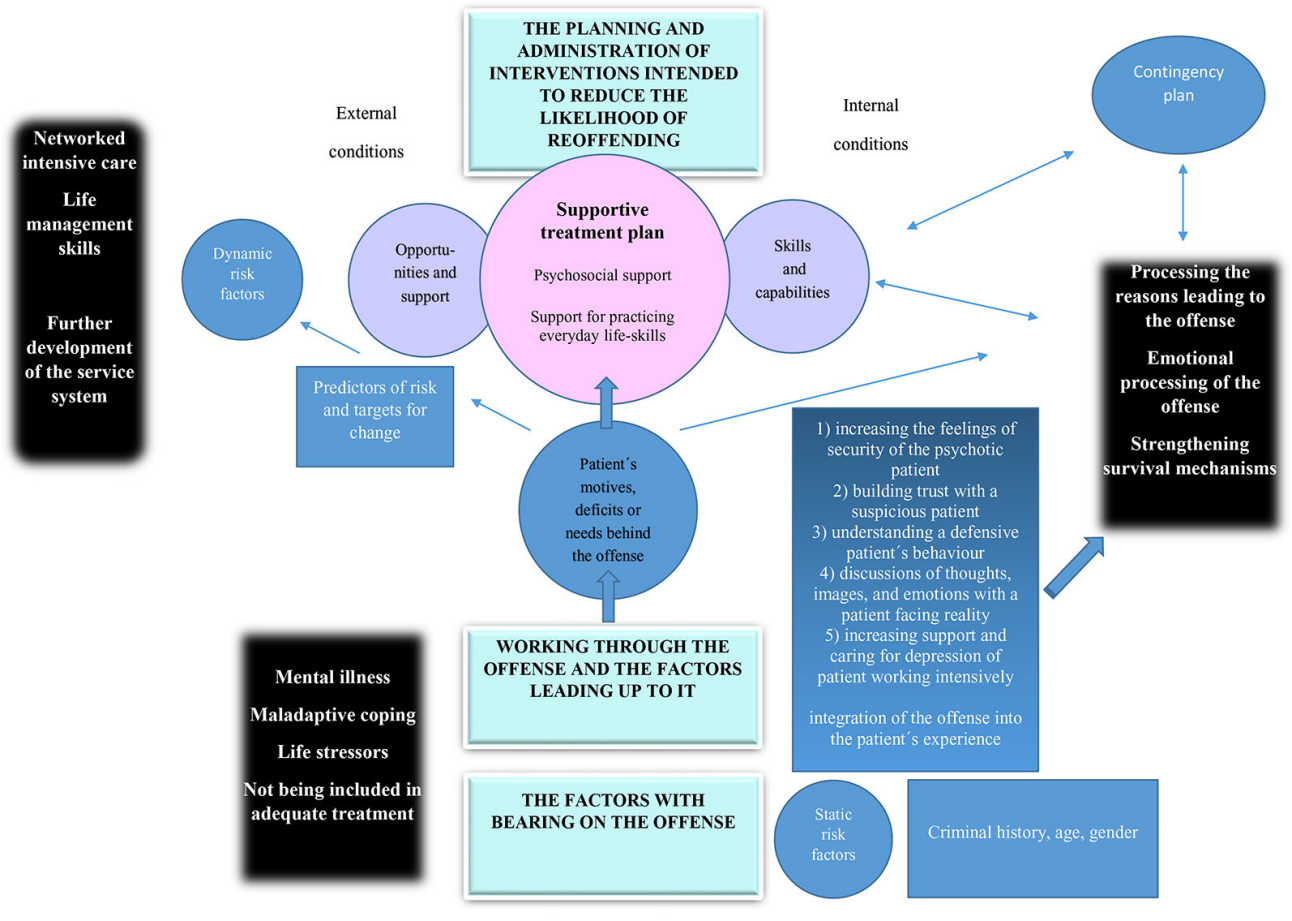
Summary of results.

The findings of this study suggest that processing the offense as a part of forensic psychiatric rehabilitation is a highly significant issue which provides support for patients’ long-term coping mechanisms. The process of working through the offense-related issues highlighted by our study must be structured around a supportive treatment plan which considers both pharmacological treatment and psychosocial support. Patients and professionals concur that an essential aspect of forensic treatment is ascertaining the factors with a bearing on the offense, working through the offense and the factors leading up to it, and the planning and implementation of interventions intended to reduce the likelihood of reoffending (**Figure 2**).

Processing the reasons leading to the commission of the offense requires great sensitivity, due to the risk of deterioration in the patient’s mental state and the potential tendency for self-harm.

The primary foci for therapeutic work on the offense are on identifying the risk factors, on the one hand, and the patient’s strengths, on the other, as conceptualized by GLM. GLM closely relates to the core principles of recovery and can be seen as a way to enhance the quality of life of patients in forensic psychiatry (41). In GLM, treatment is individually tailored to assist an offender in implementing his or her good lives intervention plan, while simultaneously addressing criminogenic needs that may be blocking goods fulfilment (29). Both patients’ and nurses’ perspectives were in line with other studies (42) in terms of developing awareness of situations that are likely to lead to offending behavior.

When striving towards a therapeutic alliance supportive enough to enable a recovery-orientated, risk-mitigating good life plan, it should be noted that the criminal act committed by the patient evokes many feelings, not only in the patient, but also in the patient’s immediate family, healthcare personnel and society in general. It is important for the personnel to scrutinize and identify their own feelings to be capable of ethically valid work with the patient and his family. Understanding that the patient committed his offense while under the influence of an illness is important to allow the relationship with the patient to be supportive, rather than punitive. Nurses face ethical decision-making situations on a daily basis when they consider the extent to which the patient is prepared to be responsible for himself or herself and to what extent his right to self-determination must be respected (43). In counselling and shared workplace values, theories of ethical decision-making (44, 45) may help when contemplating a shared value base. It becomes necessary to weigh up how to consider not only medical indications, but also factors contributing to the patient’s quality of life. These comprise understanding how to support the patient’s own choices, how to regard his or her social context such as family situation, spiritual needs, finances and the existing guidelines of the organizations and legislation (45, 46). The ethical principles of Beauchamp and Childress (44), such as support for autonomy, the right to self-determination, doing no harm, and producing good in treatment, also support the implementation of GLM.

Furthermore, to achieve the necessary balance between risk management and strength enhancement as prescribed by GLM, both internal capabilities and external conditions must be developed throughout the rehabilitative process. In terms of internal capabilities, various interventions already in established use in forensic psychiatry can be used in a targeted and individual manner (47–49). CBT based approaches, group and individual, focusing on problem-solving and interpersonal skills, have the best evidence base for the treatment of forensic patients and should be preferred to other models (1). Psychoeducation is an attempt to bring the patient to understand his or her illness better and so to motivate him to comply with his medication (48, 50), which in turn supports the goals of improving quality of life (51).

In terms of external conditions, the support afforded by intense networked care is indispensable for the forensic psychiatric patient. After a lengthy stay in institutional care, the patient encounters unfamiliar challenges in everyday non-hospital life which must be met by support measures aimed at the living environment, support for functional ability and its maintenance, and support for coping independently (52, 53). A difficult financial situation, restless living environment, or difficulty in navigating public services may constitute as big an obstacle to the patient’s rehabilitation as the psychotic illness itself. In addition to timely treatment of mental adversity and illness, there is a need for investment in the availability of welfare services, rendering them easily accessible and appropriate. The patients who participated in the present study expressed a desire for improvement in treatment commencing at a sufficiently early stage, for contact after hospital treatment, for information transfer and for a clearer division of responsibility for treatment. Accordingly, Jennings (54) recommended the provision of extended residential treatment, with a focus on life skills and treatment continuity, prior to implementing Assertive Community Treatment (ACT). According to Jennings (54), the provision of enriched or extended residential treatment—in which forensic patients have adequate time to learn, practise and master life management skills—can maximize the effectiveness of follow-up ACT.

Unfortunately, the present study indicates that the external conditions provided by general psychiatric services fall short of providing the necessary support needed to prevent the most risk-prone psychotic patients from progressing to the forensic services. The interviewees of this study brought up organizational shortcomings such as delays in treatment, being passed from one place to another and being referred to the wrong place for treatment. Most forensic psychiatric patients had been undergoing psychiatric treatment before committing their index offense. Eight out of ten forensic patients are known to have had at least once previous psychiatric hospitalization; almost half of them had been treated for substance abuse (55). In the case of some high-risk patients, the treatment and service system had failed in such a way that the patient’s high risk had not been identified or the patient had not been offered adequate and sufficiently supportive or proficient treatment. The interviews showed that not all patients had committed their offenses while suffering from fulminant, acute psychotic symptoms, but that there were other predisposing factors such as antisocial behavior, a downward spiral of escalating offenses, difficulty of social control and absent or inaccessible support.

In Finland, as elsewhere in Western Europe, the number of psychiatric beds has decreased in recent decades (56–58). However, it has been argued that this process of deinstitutionalization has not served well the needs of the most vulnerable psychiatric patient population, namely those who have the highest prevalence of risk factors for disengaging from outpatient treatment: comorbid substance misuse, high unemployment, poor social networks and a history of offenses (57). At the same time, increasing numbers of forensic patients (58) and psychotic prisoners (59) have given rise to the notion of transinstitutionalization, rather than deinstitutionalization, further increasing the misgivings—often shared by service users (60)—towards inadequate psychiatric service provision.

## Strengths and Limitations of the Research

This study investigated both the perspectives of those receiving and providing forensic mental health services, after which a synthesis of these views was generated. Traditionally, such an integrated approach has been unusual in forensic psychiatric research, perhaps due to an assumption of incompatible discordancy between views by patients and staff. Our study does not support this preconception; rather, we found that both staff and patients were concerned with similar offense-related issues, and thus a mutually inclusive thematic map emerged naturally from the interviews.

We consider our findings to represent authentic experiences of eight forensic psychiatric patients and eight nurses. However, the method of thematic interview entails, by nature, an element of reflexivity (61), as intersubjectivity and the impact of the researcher on the results is inevitable. Yet we maintain that prioritizing engagement with the interviewees over absolute scientific distance and objectivity is justified in order to support the emergence of the interviewees own voices, and the maintenance of their narrative identity and agency.

A limitation of this study is that it was conducted in two forensic settings in Finland. Therefore, the results may not be representative of all international forensic settings. A further limitation of this study concerns the small sample size and the sample demographic, which may reduce the generalizability of the findings. Moreover, the recruitment process may have not reached every patient interested in the study, as the recruitment of the patients was dependent on the nurses´ evaluation of appropriate patients. Also, our sample consisted of individuals that were willing to participate and this willingness might have skewed the sample; people disinterested in research might have different perspectives from those presented in this study.

## Conclusions

Our results suggest that patients and professionals concur that an essential aspect of forensic treatment is ascertaining the factors with a bearing on the offense, working through the offense and the factors leading up to it, and the planning and implementation of interventions intended to reduce the likelihood of reoffending by increasing patients’ quality of life. These themes should be borne in mind when developing rehabilitation programs for forensic psychiatric patients; they can serve as an aid to developing both the unique therapeutic alliance necessary for individualized rehabilitative progress and to unify evidence-based treatment models for clinical forensic psychiatric practices and services.

## Future Directions

The further development of rehabilitation programs for forensic psychiatric patients should holistically consider the main themes emerging from the interviews. With the help of these efforts, it will be possible to strengthen the patient’s protective factors, enhance self-knowledge, motivation and problem-solving ability, and to take account of predictors of risk and targets for change. A supportive and risk-aware treatment plan considers both psychosocial and pharmacological support and the practice of everyday life skills. The GLM approach can serve as a platform for developing international forensic rehabilitation standards as forensic psychiatry strives towards an increasingly firm evidence base. However, in forensic psychiatry, as in other fields of medicine, prevention is the most humane and cost-effective form of intervention. Accordingly, we support the further development of an inclusive, respectful and—when necessary—assertive general psychiatric service provision.

## Data Availability Statement

All datasets generated for this study are included in the article/supplementary material.

## Ethics Statement

The studies involving human participants were reviewed and approved by Ethics Committee of the Hospital District of Helsinki and Uusimaa. The patients/participants provided their written informed consent to participate in this study and to publish their data.

## Author Contributions

RA, PS, and AS contributed to the conception and design of the study. RA acquired and analyzed the data. RA, PS, and AS wrote the initial draft of the manuscript. All authors have contributed to, read and approved the final version of the manuscript.

## Funding

This study was supported by Helsinki University Hospital, Psychiatry.

## Conflict of Interest

The authors declare that the research was conducted in the absence of any commercial or financial relationships that could be construed as a potential conflict of interest.
